# Reasons for and against receiving influenza vaccination in a working age population in Japan: a national cross-sectional study

**DOI:** 10.1186/1471-2458-13-647

**Published:** 2013-07-12

**Authors:** Tsubasa Iwasa, Koji Wada

**Affiliations:** 1 Saiwai Public Health Center, Kawasaki, 1-11-1 Todehonmachi, Saiwai-ku Kawasaki, Kanagawa 212-8570, Japan; 2 Department of Public Health, Kitasato University School of Medicine, 1-15-1 Kitasato, Minami-ku, Sagamihara, Kanagawa 252-0374, Japan

**Keywords:** Influenza vaccination, Attitude, General population, Japan

## Abstract

**Background:**

To improve influenza vaccination coverage in the working age population, it is necessary to understand the current status and awareness of influenza vaccination. This study aimed to determine influenza vaccination coverage in Japan and reasons for receiving the vaccine or not.

**Methods:**

An anonymous internet-based survey was performed in September 2011. Our target study size was 3,000 participants between 20 and 69 years of age, with approximately 300 men and 300 women in each of five age groups (20–29, 30–39, 40–49, 50–59, and 60–69). We asked the history of influenza vaccine uptake in the previous year, and reasons for having vaccination or not.

**Results:**

There were 3,129 respondents, of whom 24.2% of males and 27.6% of females received influenza vaccination between October 2010 and March 2011. Among those who were vaccinated, the main reasons for receiving the influenza vaccine were “Wanted to avoid becoming infected with influenza virus” (males: 84.0%; females: 82.6%) and “Even if infected with influenza, wanted to prevent the symptoms from becoming serious” (males: 60.7%; females: 66.4%). Among those not vaccinated, the most frequent reasons for not receiving the influenza vaccine included “No time to visit a medical institution” (males: 32.0%; females: 22.4%) and “Unlikely to become infected with influenza” (males: 25.1%; females: 22.7%).

**Conclusions:**

The reasons for receiving the influenza vaccine varied between age groups and between sexes. To heighten awareness of influenza vaccination among unvaccinated working age participants, different intervention approaches according to sex and age group may be necessary.

## Background

Influenza vaccination is an effective preventive measure [1], and is recommended for all individuals 6 months of age or older in the United States [2]. Although influenza vaccination of healthy working adults may not be cost-saving [3], influenza vaccination can reduce the proportion of people developing influenza-like illness, the number of lost work days, and physician visits during the influenza epidemic season [4]. In the United States, where adults are encouraged to receive influenza vaccination, influenza vaccination coverage was 35.8% for individuals 18–49 years old and 51.0% for those 50–64 years old [5]. Influenza vaccination among working age Japanese people is also optional as in the United States and whether to be vaccinated or not is left to the individual. Influenza vaccination coverage in the working population of Japan [6] was approximately 30% before the influenza A (H1N1) pandemic in 2009.

Increasing uptake of influenza vaccination is an important issue to be addressed worldwide [7-9]. To increase influenza vaccination coverage, it is important to understand the motivation for and barriers against influenza vaccination uptake. Reasons for being vaccinated have been reported in previous studies, and include: the presence of chronic disease [10,11]; perceived susceptibility to influenza with a desire to avoid contracting seasonal influenza; knowing someone who became ill from seasonal influenza; protecting oneself from illness; protecting close relatives by receiving the vaccine [12,13]; reducing transmission to others [14]; being advised by a family doctor/nurse to be vaccinated [15,16]; having knowledge that influenza is a serious illness [16,17]; and having knowledge of the national vaccination strategy [18]. These reasons varied by age and sex. Reasons for not being vaccinated against influenza include: believing that influenza vaccine was unnecessary [19,20]; being concerned about side effects of vaccination [12,14,19]; believing oneself to be unlikely to contract influenza [16,17]; being unconcerned about influenza [21]; and lack of convenient access to vaccination [22].

We consider it necessary to understand the current status and awareness of influenza vaccination in order to promote influenza vaccination uptake among working age people in Japan. The aim of this study was to investigate the history of influenza vaccination in 2010 in Japanese men and women of working age (20–69 years), and to identify reasons for receiving or not receiving this vaccine.

## Methods

This study recruited 3,000 Japanese individuals aged 20–69 years who were registered by a web survey company in September 2011. People who were interested in taking part in a survey, with financial incentives for responding, registered voluntarily on the company website. The company randomly selected 7,937 subjects from a total of 1.60 million registrants based on the company protocol considering the estimated response rate to recruit 3000 participants within a week, and sent out invitations by e-mail to take part in the survey.

With regard to sample size, we determined 267 sample size needed per each gender (men and women) and 10 year age band (20–29, 30–39, 40–49, 50–59, and 60–69). based on the assumption as follows: confidence level 95%, margin of error 6, and 7 million population based on the Japanese population structure. Then, we decided to recruit 300 sample size in each gender and 10 year age band as a stratified random sampling method. The company ceased recruitment in each category once the desired number agreed to participate. We obtained agreement and consent of all subjects for participation in the survey when they completed their answers.

This study was approved by the Kitasato University School of Medicine Ethics Committee prior to implementation.

We asked participants if they had received an influenza vaccination in the period from October 2010 to March 2011, offering three possible responses: yes, no, and do not remember. Questions were formulated after reviewing previous studies [12-22] and are shown in Table 1.

**Table 1:** Questions asked in this study

I.	Of those who had received an influenza vaccination, we inquired about reasons for receiving vaccination (allowing each respondent to give as many answers as they wished	
	1.	Wanted to avoid becoming infected with influenza virus.
	2.	Even if infected with influenza, wanted to prevent the symptoms from becoming serious.
	3.	Living with family members at high risk of influenza becoming serious such as children, the elderly or pregnant women.
	4.	Received financial assistance for vaccination.
	5.	At high risk of becoming infected with influenza.
	6.	Employer ordered the vaccination.
	7.	At high risk of influenza symptoms becoming serious if infected.
	8.	Family, friends, and acquaintances recommended it.
	9.	Family doctor recommended it.
II.	Of those who had not received an influenza vaccination, we asked the reasons for not being vaccinated (multiple choices were allowed).	
	1.	No time to visit a medical institution.
	2.	Believed oneself unlikely to be infected with influenza.
	3.	Could not afford vaccination.
	4.	Lack of confidence that influenza vaccinations are effective.
	5.	Believed that disease would not likely become severe even if infected with influenza.
	6.	Concerned about adverse reactions that might occur with vaccinations.
	7.	Dislike of injections.
	8.	Lack of knowledge about where to be vaccinated.
	9.	Prior experience of an adverse reaction after being vaccinated for influenza or another disease.

### Statistical analysis

We conducted univariate analysis using Pearson’s chi-squared test to examine the potential relationships between men and women in the rate of vaccination uptake. All analyses were performed using IBM SPSS Statistics v. 19 (IBM Corp, Armonk, NY, USA), with statistical significance set at p < 0.05.

## Results

A total of 3,129 participants participated in the survey (response rate:39.4%). We recruited approximately equal numbers of males and females, and participants were relatively equally distributed among the age groups (Table 2). With respect to influenza vaccination, 24.2% of males and 27.7% of females had been vaccinated against influenza (Table 3). A slightly higher proportion of females were vaccinated than males, but there were no significant differences in the vaccination rates in any age group. The age group with the highest proportion of vaccination was aged 60–69, with 28.3% of males and 30.4% of females in this age group vaccinated.

**Table 2:** Characteristics of participants

	**N = 3129**	**(%)**
Sex		
Male	1,572	(50.2)
Female	1,557	(49.8)
Age (years)		
20-29	510	(16.3)
30-39	659	(21.1)
40-49	647	(20.7)
50-59	601	(19.2)
60-69	712	(22.8)

**Table 3:** Vaccination coverage in the year up to September 2011 by sex and age (%)

	**Male**	**Female**	
**Age group (years)**	**Number vaccinated (% of total age group)**	**Number vaccinated (% of total age group)**	**p-value**
20-29	54/262 (20.6)	68/248 (27.4)	0.19
30-39	80/332 (24.1)	93/327 (28.4)	0.35
40-49	79/326 (24.2)	75/321 (23.4)	0.86
50-59	68/302 (22.5)	85/299 (28.4)	0.21
60-69	99/350 (28.3)	110/362 (30.4)	0.69
Total	380/1572 (24.2)	431/1557 (27.7)	0.90

The reported reasons for receiving influenza vaccination are shown in Table 4. The major reasons in descending order were “Wanted to avoid becoming infected with influenza virus” (84.0% of males and 82.6% of females vaccinated); “Even if infected with influenza, wanted to prevent the symptoms from becoming serious” (60.7% of males and 66.4% of females); “Living with family members at high risk of influenza becoming serious, such as children, the elderly or pregnant women” (18.4% of males and 31.5% of females); and “Received financial assistance for vaccination” (18.6% of males and 21.0% of females). A large proportion of females aged 30–39 years reported “Living with family members at high risk of influenza becoming serious, such as children, the elderly or pregnant women” (52.8%), and many males and females aged 20–29 years gave the response that “Family, friends, and acquaintances recommended it” (20.4% of males and 16.5% of females).

**Table 4:** Reasons for having influenza vaccination(%)

	**Male**						**Female**					
**Choices**	**Total**	**20-29**	**30-39**	**40-49**	**50-59**	**60-69**	**Total**	**20-29**	**30-39**	**40-49**	**50-59**	**60-69**
	**(n = 380)**	**(n = 54)**	**(n = 80)**	**(n = 79)**	**(n = 68)**	**(n = 99)**	**(n = 431)**	**(n = 68)**	**(n = 93)**	**(n = 75)**	**(n = 85)**	**(n = 110)**
Wanted to avoid becoming infected with influenza virus.	84.0	83.8	87.8	88.5	84.6	77.1	82.6	84.0	84.2	84.5	80.3	80.9
	(80.3-87.7)	(74.0-93.6)	(80.6-95.0)	(81.5-95.5)	(76.0-93.2)	(68.8-85.4)	(79.0-86.2)	(75.3-92.7)	(76.8-91.6)	(76.3-92.7)	(71.8-88.8)	(73.6-88.2)
Even if infected with influenza, wanted to prevent the symptoms from becoming serious.	60.7	52.2	61.3	66.5	62.3	58.9	66.4	40.0	71.1	73.2	72.4	69.5
	(55.8-65.6)	(38.9-65.5)	(50.6-72.0)	(56.1-76.9)	(50.8-73.8)	(49.2-68.6)	(61.9-70.9)	(28.4-51.6)	(61.9-80.3)	(63.2-83.2)	(62.9-81.9)	(60.9-78.1)
Living with family members at high risk of influenza becoming serious	18.4	5.8	33.7	27.3	15.1	7.8	31.5	21.1	52.8	38.3	25.2	20.3
	(14.5-22.3)	(0.0-12.0)	(23.3-44.1)	(17.5-37.1)	(6.6-23.6)	(2.5-13.1)	(27.1-35.9)	(11.4-30.8)	(42.7-62.9)	(27.3-49.3)	(16.0-34.4)	(12.8-27.8)
Received financial assistance for vaccination.	18.6	3.6	22.3	27.9	15.9	18.3	21.0	13.3	21.5	23.4	22.3	22.7
	(14.7-22.5)	(0.0-8.6)	(13.2-31.4)	(18.0-37.8)	(7.2-24.6)	(10.7-25.9)	(17.2-24.8)	(5.2-21.4)	(13.2-29.8)	(13.8-33.0)	(13.5-31.1)	(14.9-30.5)
At high risk of becoming infected with influenza.	13.1	16.1	19.0	15.2	7.7	8.6	12.6	13.8	13.2	8.9	14.6	12.2
	(8.3-14.7)	(6.3-25.9)	(10.4-27.6)	(7.3-23.1)	(1.4-14.0)	(3.1-14.1)	(9.5-15.7)	(5.6-22.0)	(6.3-20.1)	(2.5-15.3)	(7.1-22.1)	(6.1-18.3)
Employer ordered the vaccination	13.9	16.9	16.9	16.6	13.7	7.8	11.1	22.3	16.2	9.5	9.8	2.0
	(10.4-17.4)	(6.9-26.9)	(8.7-25.1)	(8.4-24.8)	(5.5-21.9)	(2.5-13.1)	(8.1-14.1)	(12.4-32.2)	(8.7-23.7)	(2.9-16.1)	(3.5-16.1)	(0.0-4.6)
At high risk of influenza symptoms becoming serious if infected.	11.5	7.6	11.1	10.4	10.5	15.7	11.2	6.8	6.4	5.7	15.7	18.0
	(8.3-14.7)	(0.5-14.7)	(4.2-18.0)	(3.7-17.1)	(3.2-17.8)	(8.5-22.9)	(8.2-14.2)	(0.8-12.8)	(1.4-11.4)	(0.5-10.9)	(8.0-23.4)	(10.8-25.2)
Family, friends, and acquaintances recommended it.	8.9	20.4	5.0	9.6	5.6	7.4	11.4	16.5	8.0	4.9	7.4	18.8
	(6.0-11.8)	(9.7-31.1)	(0.2-9.8)	(3.1-16.1)	(0.1-11.1)	(2.2-12.6)	(8.4-14.4)	(7.7-25.3)	(2.5-13.5)	(0.0-9.8)	(1.8-13.0)	(11.5-26.1)
Family doctor recommended it.	7.9	3.3	5.7	2.8	6.2	17.6	6.8	1.4	2.0	3.0	7.3	16.5
	(5.2-10.6)	(0.0-8.1)	(0.6-10.8)	(0.0-6.4)	(0.5-11.9)	(10.1-25.1)	(4.4-9.2)	(0.0-4.2)	(0.0-4.8)	(0.0-6.9)	(1.8-12.8)	(9.6-23.4)

Table 5 shows the reported reasons for not being vaccinated against influenza. In males, “No time to visit a medical institution” was the most frequent reason among those aged 20–59 (32.3-42.8%), whereas “Believed oneself unlikely be infected with influenza” was the most frequent reason among those aged 60–69 (39.2%). In contrast, among females, the most frequent reasons for not being vaccinated were “No time to visit a medical institution” in those aged 20–29 (33.9%), “Could not afford vaccination ” in those aged 30–39 (35.2%) and 40–49 (28.6%), “Concern about adverse reactions that might occur with vaccinations” in those 50–59 (22.7%), and “Lack of confidence that influenza vaccinations are effective” in those aged 60–69 (31.0%).

**Table 5:** Reasons for not having influenza vaccination (%)

	**Male**						**Female**					
**Choices**	**Total**	**20-29**	**30-39**	**40-49**	**50-59**	**60-69**	**Total**	**20-29**	**30-39**	**40-49**	**50-59**	**60-69**
	**(n = 1143)**	**(n = 197)**	**(n = 242)**	**(n = 231)**	**(n = 226)**	**(n = 247)**	**(n = 1113)**	**(n = 174)**	**(n = 231)**	**(n = 245)**	**(n = 213)**	**(n = 250)**
No time to visit a medical institution.	32.0	42.8	33.7	36.6	32.3	17.3	22.4	33.9	23.5	25.9	21.0	11.1
	(29.3-34.7)	(35.9-49.7)	(27.7-39.7)	(30.4-42.8)	(26.2-38.4)	(12.6-22.0)	(20.0-24.8)	(26.9-40.9)	(18.0-29.0)	(20.4-31.4)	(15.5-26.5)	(7.2-15.0)
Believed oneself unlikely to be infected with influenza	25.1	26.1	15.3	19.5	25.1	39.2	22.7	24.8	22.4	19.8	19.3	27.1
	(22.6-27.6)	(20.0-32.2)	(10.8-19.8)	(14.4-24.6)	(19.4-30.8)	(33.1-45.3)	(20.2-25.2)	(18.4-31.2)	(17.0-27.8)	(14.8-24.8)	(14.0-24.6)	(21.6-32.6)
Could not afford vaccination.	20.1	22.7	19.2	26.7	20.2	12.4	23.8	28.4	35.2	28.6	18.2	10.4
	(17.8-22.4)	(16.9-28.5)	(14.2-24.2)	(21.0-32.4)	(15.0-25.4)	(8.3-16.5)	(21.3-26.3)	(21.7-35.1)	(29.0-41.4)	(22.9-34.3)	(13.0-23.4)	(6.6-14.2)
Lack of confidence that influenza vaccinations are effective	19.0	7.5	20.0	21.4	18.9	24.9	22.2	10.5	20.4	23.3	22.0	31.0
	(16.7-21.3)	(3.8-11.2)	(15.0-25.0)	(16.1-26.7)	(13.8-24.0)	(19.5-30.3)	(19.8-24.6)	(5.9-15.1)	(15.2-25.6)	(18.0-28.6)	(16.4-27.6)	(25.3-36.7)
Believed that disease would not likely become severe even if infected with influenza	19.3	15.2	13.6	17.0	19.0	30.6	17.5	14.3	14.4	17.0	22.0	19.4
	(17.0-21.6)	(10.2-20.2)	(9.3-17.9)	(12.2-21.8)	(13.9-24.1)	(24.9-36.3)	(15.3-19.7)	(9.1-19.5)	(9.9-18.9)	(12.3-21.7)	(16.4-27.6)	(14.5-24.3)
Concerned about adverse reactions that might occur with vaccinations	12.3	10.1	11.6	12.3	11.6	15.6	18.0	8.7	11.9	15.5	22.7	28.5
	(10.4-14.2)	(5.9-14.3)	(7.6-15.6)	(8.1-16.5)	(7.4-15.8)	(11.1-20.1)	(15.7-20.3)	(4.5-12.9)	(7.7-16.1)	(11.0-20.0)	(17.1-28.3)	(22.9-34.1)
Dislike of injections	13.9	13.5	16.3	15.9	10.7	12.8	14.1	19.2	12.9	13.2	11.1	15.1
	(11.9-15.9)	(8.7-18.3)	(11.6-21.0)	(11.2-20.6)	(6.7-14.7)	(8.6-17.0)	(12.1-16.1)	(13.3-25.1)	(8.6-17.2)	(9.0-17.4)	(6.9-15.3)	(10.7-19.5)
Lack of knowledge about where to be vaccinated	6.1	9.1	6.5	3.3	5.6	6.6	3.4	8.0	5.2	1.8	1.0	2.1
	(4.7-7.5)	(5.1-13.1)	(3.4-9.6)	(1.0-5.6)	(2.6-8.6)	(3.5-9.7)	(2.3-4.5)	(4.0-12.0)	(2.3-8.1)	(0.1-3.5)	(0.0-2.3)	(0.3-3.9)
Prior experience of an adverse reaction after being vaccinated for influenza or another disease	2.1	1.4	2.6	1.7	3.1	1.7	3.6	2.0	3.2	5.1	3.5	3.9
	(1.3-2.9)	(0.0-3.0)	(0.6-4.6)	(0.0-3.4)	(0.8-5.4)	(0.1-3.3)	(2.5-4.7)	(0.0-4.1)	(0.9-5.5)	(2.3-7.9)	(1.0-6.0)	(1.5-6.3)

## Discussion

This study aimed to determine the current status of influenza vaccination uptake in a working population (20–69 years) in Japan, and reasons for this population receiving or not receiving the vaccine. Overall, the most frequent reasons for receiving the vaccine were the desire to avoid infection with the influenza virus, and the desire to prevent symptoms becoming serious if already infected with the virus. The primary reasons for not receiving the vaccine included no time to visit a medical institution, the belief of being unlikely to become infected with influenza, and the inability to afford the vaccine, although there were variations in reasons according to sex and age.

Prevention of the onset of influenza and preventing influenza symptoms from becoming serious were the two major reasons given by individuals who received influenza vaccination. The influenza vaccine provides modest protection against the onset of influenza, with the efficacy rate reportedly being 51-67% in individuals aged 18–65 years [23]. Furthermore, 33 healthy adults need to be vaccinated to avoid one incurring influenza symptoms [24]. Twenty one percent of respondents chose “Lack of confidence that influenza vaccinations are effective” as a reason not to be vaccinated. They may be rightly critical of vaccine efficacy because of possible mismatching between circulating virus strains and the strains in the vaccine itself. The limitations of vaccine efficacy should be communicated to the general public, to maintain realistic expectations of the vaccine [15] .

The reasons for receiving influenza vaccination varied according to sex and age. Among those aged 30–39 years, a substantial proportion reported that they were vaccinated because they were living with family members at high risk of influenza becoming serious, such as children, the elderly, or pregnant women. Maternal influenza immunization is a strategy with substantial benefits for both mothers and infants [25], reflecting the fact that people aged 30–39 are commonly rearing children. Among those aged 20–29 years, a relatively higher proportion cited recommendations from family, friends, or acquaintances as the reason for accepting the influenza vaccine. These results suggest that educational messages should aim to address a wide range of possible concerns, and to improve targeted outreach to specific groups of workers.

The reasons for not receiving influenza vaccination also varied according to sex and age group. Among men aged 20–59 years, the most frequent reason for not receiving the vaccine was lack of time to visit a medical institution. A lack of time has also been cited as a major reason for healthcare workers not receiving the influenza vaccine [22]. There appear to be various misconceptions that stop people from perceiving vaccination as an important measure and one that deserves priority over other matters in their daily lives. There have been some measures that can ensure access to vaccination such as providing vaccination in pharmacies [26], and an incentive for vaccination, an intensified advertising campaign, and offering a choice of influenza vaccines can improve vaccination rates in the workplace [27]. Among men aged 60–69 years, the main reasons for not being vaccinated were the belief that they would not be infected with the influenza virus and that the disease would not become severe[28]. In Japan, influenza vaccination is recommended for people 65 years of age or older, and some local governments are providing financial support for vaccination [29]. Although men aged 60–69 may believe that they will not be infected or become seriously ill with influenza based on their experience, they should be given accurate information about the risk of infection, which increases with age.

The reasons for not receiving influenza vaccination varied more widely according to age among women than among men. The most frequent reason for not being vaccinated was lack of time to visit a medical institution in women aged 20–29, not being able to afford vaccination in those aged 30-49[30], concerns about adverse reactions in those aged 50–59, and doubts about vaccine efficacy in those aged 60–69. The avoidance of influenza vaccination among women aged 50–69 may be attributable to unfavorable views related to changes in influenza vaccination policy as a result of severe side effects and lawsuit judgments for compensation at the time their children were vaccinated [31]. If these women retain negative impressions of influenza vaccination after reaching the age of 65 when vaccination is recommended, it may be difficult to increase the influenza vaccination rate in this age group.

This study was limited because all study participants were internet users, thus its generalizability to the wider population in Japan may be restricted. It is possible that there are differences in educational status and income between internet users and non-users. In particular, internet users aged 60 or older may be better at assimilating information than are the general population. Another limitation is that because each individual chose multiple choices for questionnaire responses, and choices were not independent, we were not able to apply chi-square analysis or other statistical analysis to determine the differences according to sex and age.

## Conclusions

This study suggests that the reasons for not accepting influenza vaccination vary according to sex and age in the Japanese working age population. We recommend using different education and intervention approaches according to sex and age to increase awareness of influenza vaccination among unvaccinated participants.

## Competing interests

The authors declare that they have no competing interests.

## Pre-publication history

The pre-publication history for this paper can be accessed here:

http://www.biomedcentral.com/1471-2458/13/647/prepub
